# Precious Stones: For Curative Wear and Other Remedial Uses, Likewise the Nobler Metals

**Published:** 1908-12

**Authors:** 


**Precious Stones: For Curative Wear and other Remedial Uses, likewise the Nobler Metals.** By W. T. Fernie, M.D. Pp. xviii., 486. Bristol: John Wright & Sons, Ltd. 1907.

Here is curious medley of fact and fable, unearthed somewhat haphazard from many a forgotten book, and adorned with many a wise saw and modern instance. Scraps of folk-lore, siftings of history, and quotations from fiction ancient and modern, all contribute to illustrate the strange imaginings of human peoples in times past as well as present.

Wilkie Collins, with his *Moonstone,* is to be found bearing testimony hard by Ben Jonson, whose *Alchymist* (1610) provides a cure for epilepsy :—

> " My meat shall come in ; in Indian shells ;
> Dishes of agate, set in gold, and studded
> With emeralds, sapphires, hyacinths and rubies ;
> The tongues of carps, dormice, and camels' heels ;
> Boiled in the spirit of Sol and dissolv'd pearl ;
> —Apicius' diet 'gainst epilepsy !
> And I will eat these broths with spoons of amber,
> Headed with diamonds and carbuncles."

Holy Writ and heathen mythology lend their aid to support the officinal preparations of the earliest London Pharmacopœias. Crystal-gazing, the practice of selling butter by the yard by the banks of the Cam, and the making of good cheese, all by some chance find a place in these pages : nothing comes amiss to the inquiring and uncritical mind of Dr. Fernie. The evils of cold-water drinking are vividly recalled by meeting with the old doggerel rhyme :—

> " Full many a man, both young and old,
> Has gone to his sarcophagus,
> By pouring water icy cold
> Adown his hot œsophagus."

Nothing comes amiss to Dr. Fernie, and he is in no wise trammelled by the limitations of his book's title. He has taken heart of grace from Pliny's remark, " Nullus est liber tam malus, ut non aliqua parte prosit," until, emboldened by the encouragement derived from his books, the author introduces himself as now

> " Talking of stones, stars, plants, of fishes, flies ;
> Playing with words, and idle similes ; "

where we will take leave of him with grateful acknowledgment for having allowed us to peep over his shoulder into a most amazing wonderhouse of superstition and phantasy.

